# The quality of geriatric rehabilitation from the patients’ perspective: a scoping review

**DOI:** 10.1093/ageing/afad032

**Published:** 2023-03-15

**Authors:** Anne L Lubbe, Marjon van Rijn, Wim G Groen, Sophie Hilhorst, George L Burchell, Cees M P M Hertogh, Margriet C Pol

**Affiliations:** Departement of Medicine for Older People, Amsterdam UMC, Vrije Universiteit Amsterdam, de Boelelaan 1117, Amsterdam, The Netherlands; Amsterdam Public Health, Aging & Later Life, Amsterdam, The Netherlands; Vivium Zorggroep, Naarden, The Netherlands; Departement of Medicine for Older People, Amsterdam UMC, Vrije Universiteit Amsterdam, de Boelelaan 1117, Amsterdam, The Netherlands; Amsterdam Public Health, Aging & Later Life, Amsterdam, The Netherlands; Faculty of Health, Center of Expertise Urban Vitality, Amsterdam University of Applied Sciences, Tafelbergweg 51, 1105 BD Amsterdam, The Netherlands; Departement of Medicine for Older People, Amsterdam UMC, Vrije Universiteit Amsterdam, de Boelelaan 1117, Amsterdam, The Netherlands; Amsterdam Public Health, Aging & Later Life, Amsterdam, The Netherlands; Vivium Zorggroep, Naarden, The Netherlands; Medical Library, Vrije Universiteit Amsterdam, Amsterdam, The Netherlands; Departement of Medicine for Older People, Amsterdam UMC, Vrije Universiteit Amsterdam, de Boelelaan 1117, Amsterdam, The Netherlands; Amsterdam Public Health, Aging & Later Life, Amsterdam, The Netherlands; Departement of Medicine for Older People, Amsterdam UMC, Vrije Universiteit Amsterdam, de Boelelaan 1117, Amsterdam, The Netherlands; Amsterdam Public Health, Aging & Later Life, Amsterdam, The Netherlands; Research Group Occupational Therapy: Participation and Environment, Faculty of Health, Center of Expertise Urban Vitality, Amsterdam University of Applied Sciences, Tafelbergweg 51, 1105 BD Amsterdam, The Netherlands

**Keywords:** geriatric rehabilitation, patient perspective, quality, scoping review, qualitative research, older people

## Abstract

**Background:**

the efficacy and outcomes of geriatric rehabilitation (GR) have previously been investigated. However, a systematic synthesis of the aspects that are important to patients regarding the quality of GR does not exist.

**Objective:**

the aim of this scoping review was to systematically synthesise the patients’ perspective on the quality of GR.

**Methods:**

we followed the Scoping Review framework and gathered literature including a qualitative study design from multiple databases. The inclusion criteria were: a qualitative study design; a geriatric population; that patients had participated in a geriatric rehabilitation programme and that geriatric rehabilitation was assessed by the patient. The results sections of the included studies were analysed using a thematic analysis approach.

**Results:**

twenty articles were included in this review. The main themes identified were: (i) the need for information about the rehabilitation process, (ii) the need for telling one’s story, (iii) the need for support (physical, psychological, social and how to cope with limitations), (iv) the need for shared decision-making and autonomy, (v) the need for a stimulating rehabilitation environment and (vi) the need for rehabilitation at home.

**Conclusion:**

in this study, we identified the aspects that determine the quality of rehabilitation from the patient’s perspective, which may lead to a more holistic perspective on the quality of GR.

## Key Points

- This review presents the first steps into identifying the range of themes/aspects that patients consider important when in geriatric rehabilitation.
- More attention to the questions and needs of the patient might ensure that rehabilitation will be better adapted to the patients’ preferences and needs.
- Future research should focus on how best to assess patients’ experiences in the different phases of rehabilitation and how care teams can use these experiences to improve the quality of geriatric rehabilitation.

## Introduction

Globally, the size of the population that are aged 65 years and older will rapidly increase in the upcoming decades and is estimated to reach over 1.5 billion in 2050 [1]. This global ageing population is associated with increased multi-morbidity and geriatric syndromes [2], which increase the likelihood of adverse health outcomes, such as functional decline, hospitalisation and mortality [3]. After hospital admission, many patients require rehabilitation to recover from their functional decline or other specific care needs [4, 5]. This has resulted in an increased demand for rehabilitation for older people, also called geriatric rehabilitation (GR) [6, 7]. Recent numbers of older patients in GR worldwide are difficult to define because of the variety in definitions and inclusion criteria. However, Marengoni *et al*. [8] mentions that 11% of those hospitalised who are 75 years and older are discharged to a geriatric rehabilitation facility. In the Netherlands, 53,000 people received geriatric rehabilitation in 2019 [9]. Geriatric Rehabilitation (GR) is defined as ‘a multidimensional approach of diagnostic and therapeutic interventions, the purpose of which is to optimize functional capacity, promote activity and preserve functional reserve and social participation in older people with disabling impairments’ [10]. Patients in GR are mostly of old age (i.e. 75 years and older), are often frail and have several comorbidities, including cognitive dysfunctions and communication problems. However, they want to achieve self-dependence after a medical event [11]. The main goals of GR often are the advancement of activities of daily living (ADL), social involvement, and participation while ensuring an increase in the quality of life and overall well-being of the patients [12, 13]. GR can be offered as a home-based service or as an inpatient care trajectory within hospitals, rehabilitation hospitals, skilled nursing facilities or nursing homes with rehabilitation units [10].

The efficacy and outcomes of GR have been well investigated [14]. There is strong evidence on the benefits of GR for patients after suffering from a stroke [15], hip fracture [16, 17] or COPD [18, 19]. For GR to succeed, coordination between the patient and the professional when setting rehabilitation goals is important. If there is a mismatch between the goals set by the patient and those set by the health care professional, goals will be more difficult to complete, optimal functioning will be harder to achieve and resources will be wasted [20–24]. Interventions to facilitate goal setting together with patients and professionals have shown positive effects on the quality of GR [12, 13]. Incorporating shared decision-making between the patient and the professional during goal setting is important for the quality of care (QoC) in GR [25] but has not been specifically investigated from the perspective of the patient. Moreover, little is known about the quality of GR as perceived by the patients themselves.

The inclusion of these perspectives is of utmost importance during the rehabilitation process, especially with regards to its effectiveness [26, 27].

The aim of this scoping review (ScR) is to systematically synthesise the quality of GR from the patient perspective. More specifically, we seek to answer the following research question: from the patients’ perspective, what is important for the quality of geriatric rehabilitation? To answer this question, we will review qualitative studies that address the quality of GR from the patient’s point of view.

## Method

### Literature review

#### Scoping review

We followed the ScR framework outlined by Arksey and O’Malley [28], refined by Levac *et al*. [29], Colquhoun *et al*. [30] and Daudt *et al*. [31]. We also adhere to the Preferred Reporting Items for Systematic Review and Meta-Analysis Protocols extension for ScRs. Compared with systematic reviews that often have very specific questions, scoping reviews are exploratory in nature and are better suited for understanding the breadth of a concept or field. Scoping reviews can inform more specific recommendations for future research [32]. The process for a scoping review includes five steps: (i) identifying the research question(s); (ii) identifying relevant literature; (iii) study selection; (iv) charting the data and (v) collecting, summarising and reporting the results [28–31].

#### Systematic literature search

A systematic search was performed across the following databases: PubMed, Embase.com, Clarivate Analytics/Web of Science Core Collection, Cumulative Index to Nursing and Allied Health Literature (CINAHL) and the Cochrane Library. The search timeframe within the databases was from inception to 20th July 2022 and was conducted by G.B.L. and A.L. The search included the following keywords and free text terms: for (synonyms of) ‘rehabilitation’ or ‘physiotherapy’ combined with (synonyms of) ‘rehabilitation centre’ or ‘nursing home’ combined with (synonyms of) ‘geriatric population’ or ‘elderly’ combined with (synonyms of) ‘quality indicators’ combined with (synonyms of) ‘patient perspective’ or ‘self-reporting’ combined with (synonyms of) ‘qualitative research’. A full overview of the search terms used for each database can be found in Appendix 1. No limitations on date or language were applied during the search.

#### Study selection

Study selection took place over two steps. In the first step, two team members (A.L. and S.H.) independently assessed the titles and abstracts relevant to the research question against the inclusion criteria. A.L. and S.H. agreed to include a study for full text screening when they could not determine its relevance based on the abstract and title alone. In the second step, potentially relevant studies were assessed on their full text by means of applying the selection criteria (A.L.). If there was uncertainty, a second team member (M.R./M.P.) was asked to review the article. In the case of disagreement, consensus was sought through additional discussions.

The inclusion criteria were:

- A qualitative study design.
- Includes a geriatric population (age).
- Patients participating in a geriatric rehabilitation program.
- Aspects of the quality of geriatric rehabilitation assessed by the patient themselves.
- Articles in either Dutch or English.

Articles were excluded if they contained:

- Patients permanently living in long-term care, without a programme for geriatric rehabilitation
- Any patient < 55 years and/or with a mean age < 60 years

#### Charting the data

A data extraction form was developed by A.L. to identify the variables from the included articles and was piloted on five articles by the reviewers (A.L., M.R. and M.P.). In the extraction form, we collected the relevant information for the scoping review, specifically the patients’ point of view regarding the quality of GR, which was divided into possible categories such as aim, patient perspective, quality of care, quality of life, key findings and GR assessment. The extraction form was completed by A.L. If there was any doubt, M.R./M.P. were consulted for consensus. The extraction form can be found in Appendix 2.

#### Collating, summarising and reporting the results

We performed a qualitative synthesis [33] to classify the information according to emerging themes. Thematic analysis was chosen as a flexible methodological approach to cluster and summarise the data retrieved from the included studies [34]. The results sections of the included studies were coded using MAXQDA2020. The first author coded the articles (open coding), while the first five articles were blind coded by AL/MR/MP. If there was any doubt on the other articles, a team member of the research group was consulted (M.R./M.P.). The initial notes and headings were grouped into a starting set of themes. The codes were iteratively discussed amongst the research group in order to obtain a final set of themes.

## Results

**Figure 1:**
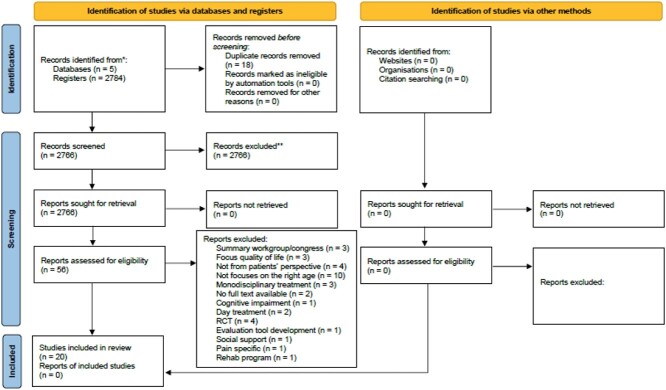
Flow chart [60].

The process of study selection is depicted in a PRISMA Flow Diagram (Figure 1). In this scoping review, the initial search yielded 2,784 articles. Out of these, 56 articles were selected for full text review and 20 studies were included in this scoping review. The full text version was not available for 2 of the articles. For other exclusions, see Figure 1.

### Characteristics of the included studies

The sample size of the participants in the studies ranged from 5 to 53 participants. Manuscripts were published across eight different countries and were published from 1999 to 2019, with 12 of the studies published after 2015. All included articles were peer-reviewed journal articles. Further information about the included studies is presented in Table 1.

**Table 1:** Study characteristics

Author, year, country	Qualitative study type	No. of patients (male/female)	Age	Disease	Aim
Archibald, 2002, UK [35]	Phenomenological ➔ interviews	5 (1/4)	>65 years	Hip fracture	To reveal participants’ experiences in order to gain insights into how to improve nursing care for people after a hip fracture.
Atwal, 2005, UK [36]	Post-discharge interviews	43 (18/25)	Male mean age = 81.4 years, Female mean age = 78.5 years	Mixed pathologies	To explore whether healthcare professionals are actively engaging older adults in rehabilitation programmes.
Chen, 2019, China [37]	Semi-structured interviews	21 (8/13)	Mean ± SD 66.28 ± 5.21 years	15 hip arthroplasty 6 hemiarthroplasty	To investigate the needs of Chinese patients undergoing home-based rehabilitation after a hip replacement.
Chen, 2015, Australia [38]	Interpretive study design	22 (12/10)	Mean age = 69 years	Stroke	To explore community-dwelling, first-time stroke survivors and family caregivers’ perceptions of being engaged in stroke rehabilitation.
Demir, 2015, Turkey [39]	Qualitative descriptive design ➔ interviews	53 (28/25)	Mean ± SD 60.79 ± 16.14 years	Stroke	(i) What are the problems faced by the patients after stroke? and (ii) What does recovery after stroke mean to the patient?
Gill, 2015, Australia [40]	Semi-structured interviews	20 (8/12)	Mean age = 75 years (45–89 years)	Orthopaedics, amputations, neurological and debilitations	To determine patient, staff and community volunteer opinions and experiences of point of service feedback (POSF) in an inpatient rehabilitation facility.
Ellis-Hill, 2009, UK [41]	Semi-structured interviews	20 (12/8)	Mean age = 70 years (53–85 years)	Stroke	What constitutes a ‘good’ or ‘poor’ experience in relation to the transition from hospital to home?
Krevers, 2009, Sweden [42]	Semi-structured interviews	14 (3/11)	Mean age = 78 year (63–91 years)	Not reported	To gain a deeper understanding of how elderly people experience and evaluate the care and rehabilitation process.
Lou, 2017, Denmark [43]	Semi-structured interviews	22 (15/7)	Male mean age = 70 years, Female mean age = 65 years	Stroke	How mild stroke patients and their partners experience and manage everyday life in the context of early supported discharge (ESD).
McKain, 2004, Australia [44]	Semi-structured interviews	9 (1/8)	Mean age = 74.9 years (54–93 years)	4 fractures, 2 CVAs, 1 prosthesis removal, 1 Guillian Barre, 1 syncope	What are the needs before transfer from an acute care facility to a rehabilitation setting that could assist patients to engage actively in rehabilitation activities upon entering the unit.
Medina-Mirapex, 2016, Spain [45]	Semi-structured interviews	15 (7/8)	Mean age = 65.6 years (SD 9.5)	4 strokes, 4 knee replacements, 7 hip replacements	To investigate rehabilitation continuity from the patients’ perspective.
Nielsen, 2018, Denmark [46]	Qualitative interviews	11 (8/3)	Mean age = 74.7 years	1 back pain, 3 infections, 2 respiratory, 4 neurological, 1 heart problems	To explore and gain a deeper understanding of elderly patients’ experiences of being discharged and their return to everyday lives after discharge from a short-stay unit in the emergency department.
Phelps, 2019, UK [47]	Semi-structured interviews	11 (2/9)	Mean age = 76.3 years (54–93 years)	Distal femoral fracture	To examine patients’ experiences during treatment and the early stages of recovery.

*(continued)*

**Table 1:** Continued

Author, year, country	Qualitative study type	No. of patients (male/female)	Age	Disease	Aim
Pol, 2019, The Netherlands [17]	Semi-structured interviews	19 (7/12)	Range 65–94 years	Hip fracture	What aspects of the recovery process after hip fracture do community-dwelling older adults perceive as the most beneficial for their return to everyday life?
Proot, 1999, The Netherlands [48]	Grounded theory study ➔ interviews	17 (10/7)	Mean age = 72.5 years (50–85 years)	Stroke	To explore patient autonomy during rehabilitation.
Proot, 1999, The Netherlands [49]	Qualitative study ➔ interviews	20 (12/8)	Mean age = 72.4 years (50–85 years)	Stroke	To determine which facilitating or constraining factors regarding patient autonomy at discharge are identified by stroke patients in nursing homes.
Proot, 2007, The Netherlands [50]	Qualitative study ➔ open-ended interviews	20 (12/8)	Mean age = 72.4 years (50–85 years)	Stroke	To explore stroke patients’ experiences of health professionals’ approach towards autonomy in a longitudinal way.
Purcell, 2018, Australia [51]	Semi-structured interviews	8 (5/3)	Mean age = 72 years (45–87 years)	Stroke	To explore stroke survivors experiences of engagement in occupations during stroke rehabilitation.
Reay, 2015, Australia [52]	Face-to-face interviews	10 (4/6)	>65 years	Total hip replacement (THR)	To describe the post-discharge experience of elderly patients following primary THR and recommend strategies to improve the discharge process.
Seben, 2018, The Netherlands [53]	Semi-structured interviews	10 (5/5)	>80 years	7 fractures, 2 cardiovascular diseases, 1 pin replacement	To gain a deeper understanding of the essence of rehabilitation goals from a patient’s perspective.

### Characteristics of the participants

The mean age of the patients varied from 60.8 to 81.4 years. There was a large variety of diagnoses across the participants; (i) four articles focused on hip fractures [17, 35, 37, 52], (ii) eight articles included stroke patients only [38, 39, 41, 43, 48–51], (iii) seven articles included a mixed sample of patients with different pathologies [36, 40, 42, 44–46, 53], (iv) one article included patients with distal femoral fractures [47] and (v) one article gave no description of the studied population [42]. Only two studies included patients with cognitive problems. The first by Chen *et al*. [38] considered post-stroke cognitive decline and the second by Phelps *et al*. [47] described one patient with dementia. All of the included studies used interviews as their main methodology.

### Thematic analysis of the findings

The open coding of the results sections of the 20 included articles led to a set of codes. These codes were grouped into an initial set of themes. This initial set of themes was discussed amongst the project group which resulted in the following themes related to the quality of GR from a patient’s perspective: (i) the need for information about the rehabilitation process, (ii) the need for telling one’s story, (iii) the need for support (physical, psychological, on how to cope with limitations and social), (iv) the need for shared decision-making and autonomy, (v) the need for a stimulating rehabilitation environment and (vi) the need for rehab in the home. These themes will be addressed below.

### The need for information about the rehabilitation process

Patients reported a lack of information throughout the rehabilitation process. Several studies find that patients experience a lack of information about the rehabilitation process at the beginning of GR [36, 38, 42, 44–50]. Furthermore, patients lacked information on and an explanation about their health status [37, 42, 45]. In another study, patients were hesitant to ask questions because they felt that the health care staff were already overwhelmed [38]. Some patients felt uncertain about their own health with the limited information they had received [38, 46]. During discharge at the end of the rehabilitation period, a number of studies indicated that patients needed more information about discharge, the process of going home and on how their lives would look in the future [41, 46, 48, 50].

### The need for telling one’s story

Letting patients tell their story is the first step in rehabilitation [35]. Being asked for their experience is very positively received by patients [40]. Telling a story to a fellow patient allows the story to be put into perspective, which has a positive effect on rehabilitation [48]. If there is more time for interaction with medical staff during in-patient rehabilitation, this allows for greater levels of trust to be built and telling one’s story [36]. Patients experience it as supportive when people listen, which helps them put their experiences into perspective. Furthermore, patients experienced frustration when they felt that they were not being heard [42]. Patients also felt limited in telling their stories. One cause of this was the high workload of healthcare staff. The patients respected this, but ideally, they would like this to be different [36].

### The need for support

Having support from professionals provided comfort and made patients feel that they had learnt about their rehabilitation. The consistent delivery of care promoted this [45]. Rehabilitation is not just about physiotherapy and explaining how to do an exercise, but it is also about motivation and promoting progress at every stage of the process, especially when patients experience bad days [47]. Support from professionals also increases patient’s self-confidence with regards to discharge, which has a positive impact on follow-up procedures [41]. Patients indicated that continuity of care was very important, as experiencing inconsistency can cause confusion [45]. The support of a professional can be supplemented with the use of eHealth applications, which enables remote support [17]. An active approach to follow-up is desirable as patients did not feel supported when they were merely informed about the possible opportunities for support [41]. The balance between professional support and autonomy means that the patient gains more control over their rehabilitation.

During rehabilitation, patients experience various limitations in their functioning and need support for this. These needs as perceived by the patients can be divided into four different categories.

#### Need for physical support

Patients experienced limitations because of what they had gone through, and these limitations had not been fully overcome after intramural rehabilitation. In the home setting, patients still experienced a lack of energy, so they had to ask for more help and required more rest. Pain and mobility limitations played a role in daily life and although physical functioning improved, these limitations remained [17, 46].

#### Need for psychological support

Psychological factors were not always included in the rehabilitation process [36]. Being confronted with activities that are no longer possible may lead to sadness or irritation [50]. Moreover, sadness could result from a long hospitalisation period in a medical and rehabilitation setting before returning home [47]. During this period of admission, unfinished tasks, such as being helped to go to the toilet by medical staff and being left there, may have had an effect on the patient’s mental health [36]. For one patient in that specific situation, it did have an impact on their psychological health [42]. During discharge and in the home setting, this traumatic incident continued to play a role in their daily functioning [17]. Furthermore, daily functioning can be influenced by thoughts about falling again [17], fear of complications [37] and unanswered questions, which made it difficult to continue getting on with life [41]. The patients experienced different kinds of losses regarding their health, activities, roles, integrity and their view of the future [33].

#### Need for support in coping with limitations

Coping can play a major role in how rehabilitation progresses [17]. For example, restrictions due to surgery, such as not putting strain on a leg, were perceived as frustrating; however, differences were noticeable across genders and ages [52]. Differences in what patients asked of themselves and what patients could functionally do led them to feeling frustrated [48]. After discharge, further frustration occurred due to the limitations on daily functioning [46]. The loss of the ability to perform daily activities could also lead to stress [17]. Patients’ frustrations required dialogue to ensure rehabilitation was a success [42]. Participants returning to their home had to adapt and develop new skills to cope with their mobility restrictions and factor in the use of unfamiliar walking aids. The participants described using various strategies to cope and adapt to their restrictions, alongside the support of health care professionals [44].

#### Need for social support

The main goal of rehabilitation for the patient is to be able to participate in society [39]. However, patients felt that their social role had changed [37], partly due to admission to a medical setting [50]. Social isolation during admission, but also after discharge, is common. Due to physical limitations, participation in social activities may be more limited for the patient [52]. Contact with others was of great importance. This included family and friends, but also fellow patients [42]. The consequences of social isolation without intervention are symptoms of depression, a higher burden on caregivers and a lower quality of life [38].

### The need for shared decision-making and autonomy

A patient’s level of autonomy changes during rehabilitation: as discharge approaches, it shifts from passive to active, whereby patients interact more with the rehabilitation process [50]. Patients may find it difficult to make decisions on their own, which highlights the importance of shared decision-making [48]. This can be accommodated by including the patient in team meetings [48]. GR helps to regain independence in ADL and to take more control, thus encouraging autonomy [53].

Preferences must be discussed in the rehabilitation process: an example of this is analysing the problems and strengths the patient has, and how to apply them to solve their issues [36]. For stroke patients, shared decision-making is of great importance [50]. Patient’s motivation can increase if they have an active role at the centre of the process, with a personal rehabilitation plan playing an important role [39].

Patients reported that shared decision-making during goal setting was very important to them. Patients involved in goal setting and planning of their rehabilitation experience reported being more satisfied with their long-term self-care and were better able to self-manage [38]. Being aware of what phase patients are in during the rehabilitation process helps when constructing a plan and assigning realistic goals for the patient [35]. The main goal for patients in rehabilitation is returning to their home environment and it is important for them that they are supported in this goal [36]. A great motivator for therapy is working towards recovery and a degree of functioning similar to life before the medical event [51]. Patients rehabilitating with the aim of reaching pre-hospitalisation functioning require functional training during therapy [42]. While the goal of going home is pursued, it may be that after discharge patients may have a desire to stay longer and to make even more progress [53]. A patient values functional skills, while the priorities of a professional are more focused on self-care. In addition, the professional also has a critical view on the feasibility of the patient’s goals [39]. From the patient’s perspective, the goals of professionals are more focused on discharge and appear to have little concern for the patient’s long-term goals [53], which highlights the need for shared decision-making. A lack of continuity in rehabilitation is a major concern for patients, as it limits goal setting across the different stages of rehabilitation [38].

### The need for a stimulating rehabilitation environment

The atmosphere in a rehabilitation setting is experienced differently than in a hospital [48]. One difference is that the entire environment of the GR setting is designed to support the rehabilitation of older people and is adapted to the patients’ functioning [41]. During in-patient rehabilitation, therapy is seen as the most important activity and this is what gives structure to life. In addition, moments of rest are also experienced as very important [51]. Fellow patients can have a major impact on the rehabilitation process, for example, conversations with others during intramural rehabilitation are considered pleasant [17]. Moreover, seeing how other patients are doing can also have a positive impact [53]. The people around the patient are important, but environment also has a large impact on other factors, such as safety and comfort [42]. The importance of support from family and friends during rehabilitation is emphasised by patients [52]. In addition to mental support, family and friends also take on other practical tasks, such as transportation to and from the hospital [37]. The environment of the rehabilitation department should promote social interaction between the patient and their family and friends. In addition to the people around a patient, culture and religion can also play an important role [37].

### Need for rehabilitation in the home setting

#### Follow-up

After discharge, patients may still need rehabilitation support, for which contact with follow-up care institutions is important. For a patient, planning and arranging contact with care and municipal services is a major task [42]. A team of care providers that come to the home after discharge eases this process for the patient [43]. Having the contact details of the rehabilitation team makes it easier to ask questions after discharge and knowing that someone is there to answer their questions could be helpful [47].

#### Rehabilitation at home

Many patients experienced discharge as very difficult [17]. A lack of information about the discharge and follow-up process was confusing [45]. Patients still need rehabilitation at home; however, the options and frequency are limited [47]. The feeling of not being ready for rehabilitation after discharge from in-patient rehabilitation has to do with coordination of the discharge. Here, a coordinating nurse could play a significant role [38]. Inadequate discharge planning may lead to patients feeling neglected [52]. Patients feel supported when they are prepared for discharge and when they work towards relevant goals [41].

#### Expectations for the future

Rehabilitation is often aimed at improving functioning, however pre-hospitalisation functioning may not yet have been achieved at the moment of discharge. If patients function at a basic ADL level, they are usually discharged and are able to function at home [53]. Some patients speak optimistically about the future, while others accept that mobility is more limited [47]. Accepting limitations is especially difficult for people who had a high level of functioning prior to admission. Some patients report that they feel like they are losing their independence [47]. Expectations after discharge are usually positive as patients expect to attain a rather similar level of functioning as before admission. However, there is also some concern about functioning, for example as to whether it is getting better and if they will achieve their level of pre-hospitalisation functioning again [46]. Not being able to do what you want to do can be frustrating, therefore, patients hope that their functioning will improve and that there will be opportunities participate again, in addition to functioning well at home [53].

## Discussion

The aim of this study was to systematically synthesise patients’ perspectives on the quality of GR. In this scoping review, 20 qualitative studies were included. Six themes resulting from this scoping review reflect the patient’s perspective as to the quality of geriatric rehabilitation care: (i) the need for information about the rehabilitation process, (ii) the need for telling one’s story, (iii) the need for support (physical, psychological, on how to cope with limitations and social), (iv) the need for shared decision-making and autonomy, (v) the need for a stimulating rehabilitation environment and (vi) the need for rehabilitation in the home setting.

Many of these themes have been found to be important in previous studies on the quality of medical rehabilitation. Giesler *et al*. [54] and Nielsen *et al*. [55] also found themes concerning insufficient information on the scope and process of rehabilitation for adults, insufficient support from physicians and family, participation in treatment decisions, experiencing physical and emotional burdens, being misunderstood and abandoned by health care professionals, a lack of understanding on the motor disorder and the need for careful communication to understand and accept the diagnosis.

Janssen *et al*. [56] examined the quality of rehabilitation from the professional’s point of view. This resulted in the following eight elements: client centeredness, client satisfaction during rehabilitation, therapeutic climate, information provision to client and informal care givers, consultation about the rehabilitation (process), cooperation within the MDT, professionalism of GR professionals and organisational aspects.

The elements defined by Janssen *et al*. [56] and the themes found in our study partly overlap, such as considerations as to the environment and the rehabilitation climate, which both professionals and patients value. Patients indicated that telling one’s story is of great importance, which is an aspect that is not mentioned by Janssen *et al*. However, it does contribute to patient satisfaction, which is considered valuable from a professional perspective [19]. The importance of information is seen as significant by both parties (caregiver and patient, but also the informal caregiver) [56]. In addition, ‘satisfaction during rehabilitation’ is postulated as an important aspect of quality by Janssen and colleagues [56], but this topic does not emerge specifically from the qualitative interview studies [19]. A comparison between the different perspectives could demonstrate the apparent variations, which can form the basis for further research. This comparison shows that a number of aspects that the patient understands by quality are already taken into account by the healthcare professional, while others are not, such as listening to a patient’s story and the need for shared decision making and autonomy.

What this study adds are elements on the quality of rehabilitation from the patient’s perspective, which can lead to a more holistic perspective on quality. This scoping review contributes an overview of the knowledge from the perspective of the patient. To date, GR has mainly been described from the perspective of the professionals. Patients should be given space to share their care experiences in a way that they feel comfortable. Focusing on meaningful care experiences as a whole can contribute to a new way of assessing the experienced quality of care [57–59]. The patient’s perspective completes our knowledge on the quality of care, and it is imperative that the patient should participate and have control in the rehabilitation process. In addition, communication and support from professionals plays an important role.

The perspectives of the geriatric patient have only been measured at one point in time in all of the included studies [17, 35–53], which is a limiting factor in the current literature. In addition, perspectives on the quality of GR may change during and after rehabilitation. Ideally, the quality of GR should be investigated and evaluated from multiple perspectives, both during and after rehabilitation: the professionals, the patients and that of the informal caregivers. This lays the foundation for further research into the patient’s perspective of quality throughout the process of geriatric rehabilitation. In possible follow-up research in which patients are followed longitudinally, this point can be addressed more specifically in order to take into account the wishes of the patients.

### Strength and limitations

A strength of this scoping review is the in-depth and comprehensive summary of qualitative studies performed on patients undergoing geriatric rehabilitation. The review of qualitative research best reflects the patient’s perspective on the quality of geriatric rehabilitation. This provides the basis for further research to increase the quality of GR, with the acknowledgement that rehabilitation is a process that could change over time. This study complements the overview on the quality of rehabilitation care from different perspectives through the addition of the patient perspective. However, this study has some limitations that might impact its generalisability.

First, the restriction of publication languages to Dutch and English could have limited the generalisability of our findings; there may be additional information written in a language other than English or Dutch. Second, we observed that in total, only two studies included participants with cognitive impairment. Therefore, the current results are not generalisable to older people with severe cognitive impairment. It is important to know how these patients think about what is important regarding the quality of GR and future research should address this remaining uncertainty. Third, we aimed to include studies on in- and outpatient rehabilitation. However, the results show that the majority of studies are conducted among patients who are treated in the inpatient setting. Therefore, the outpatient setting also should be further studied. Finally, 8 of the 20 included studies are about stroke patients, making the results less generalisable to the entire GR population.

The information obtained is from summarised findings of qualitative studies and not from the primary data, which can be seen as a limitation. However, to create a concise overview of the data, we did not think it was necessary to use the original data.

## Conclusion

This scoping review identified six themes reflecting the patient’s perspective as to the quality of geriatric rehabilitation: (i) the need for information about the rehabilitation process, (ii) the need for telling one’s story, (iii) the need for support (physical, psychological, on how to cope with limitations and social), (iv) the need for shared decision-making and autonomy, (v) the need for a stimulating rehabilitation environment and (vi) the need for rehabilitation in the home setting.

Involving the patient perspective when defining the quality of GR may improve the experienced quality of GR for both patients and health care professionals, thus contributing to a more holistic perspective on quality.

This review presents the first steps into identifying the range of themes/aspects that patients consider important in GR. To achieve a high-quality care experience in rehabilitation, future research should focus on how to best assess patients’ experiences in the different phases of rehabilitation, on including people with cognitive impairment, on the outpatient setting and on how care teams can use these experiences to improve the quality of GR.

## Supplementary Material

aa-22-1755-File002_afad032

aa-22-1755-File003_afad032

## Data Availability

The data will be made available from the corresponding author on reasonable request.
