# Supplementary material for: The quality of geriatric rehabilitation from the patients’ perspective: a scoping review
Source: Age Ageing. 2023 Mar 15;52(3):afad032. doi: 10.1093/ageing/afad032 (PMC10020894; doi:10.1093/ageing/afad032)
Supplement: aa-22-1755-File002_afad032 [file aa-22-1755-file002_afad032.docx]

## The quality of geriatric rehabilitation from the patients’ perspective: a scoping review.

## Appendix 1

## Table 1: Search strategy in PubMed

| **Search** | **Query** | **Results** |
| --- | --- | --- |
| **#7** | **#1 AND #2 AND #3 AND #4 AND #5 AND #6** | **1,213** |
| **#6** | **"Qualitative Research"[Mesh] OR "Focus Groups"[Mesh] OR "Interview" [Publication Type] OR "Interviews as Topic"[Mesh] OR "Narration"[Mesh] OR "Personal Narratives as Topic"[Mesh] OR "Observational Studies as Topic"[Mesh] OR "Observational Study"[Publication Type] OR "Tape Recording"[Mesh] OR "Grounded Theory"[Mesh] OR "thematic analys*"[tiab] OR "content analys*"[tiab] OR "focus group*"[tiab] OR "ethnograph*"[tiab] OR "ethnograf*"[tiab] OR "etnograf*"[tiab] OR "field stud*"[tiab] OR "phenomenolog*"[tiab] OR "narration*"[tiab] OR "narrative"[tiab] "case stud*"[tiab] OR "qualitative stud*"[tiab] OR "qualitative analys*"[tiab] OR "qualitative research*"[tiab] OR "qualitative method*"[tiab] OR "multimethodolog*"[tiab] OR "mixed method*"[tiab] OR "observation*"[tiab] OR "grounded theor*"[tiab] OR "audio recording*"[tiab] OR "tape recording*"[tiab] OR "audiotape*"[tiab] OR (("semi-structured"[tiab] OR "semistructured"[tiab] OR "unstructured"[tiab] OR "informal"[tiab] OR "in-depth"[tiab] OR "indepth"[tiab] OR "face-to-face"[tiab] OR "structured"[tiab] OR "guide*"[tiab]) AND ("interview*"[tiab] OR "discussion*"[tiab] OR "questionnaire*"[tiab]))** | **1,198,178** |
| **#5** | **"patient perspective*"[tiab] OR "patient s perspective*"[tiab] OR "patient centred" OR "patient centered" OR "experience*"[tiab] OR "belief*"[tiab] OR "satisf*"[tiab] OR "attitude*"[tiab] OR "expectation*"[tiab] OR "reaction*"[tiab] OR "opinion*"[tiab] OR "acceptance"[tiab] OR "view*"[tiab] OR "perception*"[tiab] OR "concern*"[tiab] OR "self report*"[tiab]** | **4,231,140** |
| **#4** | **"Quality Indicators, Health Care"[Mesh] OR "quality indicator*"[tiab] OR "Health Metric*"[tiab] OR "quality of rehabilitation*"[tiab] OR "function activity and participation"[tiab] OR "international classification of functioning"[tiab] OR "ICF model"[tiab] OR "Quality of Life"[Mesh] OR "Activities of Daily Living"[Mesh] OR "Health Status"[Mesh] OR "quality of life"[tiab] OR "life qualit*"[tiab] OR "living qualit*"[tiab] OR "quality of living"[tiab] OR "activities of daily living"[tiab] OR "activity of daily living"[tiab] OR "activities of daily life"[tiab] OR "activity of daily life"[tiab] OR "daily living activit*"[tiab] OR "daily life activit*"[tiab] OR "adl"[tiab] OR "chronic limitation of activity"[tiab] OR "self care*"[tiab] OR "health status"[tiab] OR "level of health"[tiab] OR "health level*"[tiab] OR "qol"[tiab] OR "hrql"[tiab] OR "hrqol"[tiab] OR "time of discharge"[tiab] OR "discharge time"[tiab]** | **679,512** |
| **#3** | **"Aged"[Mesh] OR "Aged, 80 and over"[Mesh] OR "Frail Elderly"[Mesh] OR "Geriatrics"[Mesh] OR "Geriatricians"[Mesh] OR "Geriatric Psychiatry"[Mesh] OR "Geriatric Nursing"[Mesh] OR "Geriatric Dentistry"[Mesh] OR "Dental Care for Aged"[Mesh] OR "Health Services for the Aged"[Mesh] OR "Housing for the Elderly" [Mesh] OR "Homes for the Aged" [Mesh] OR "Nursing Homes"[Mesh] OR "Senior Centers"[Mesh] OR "Retirement"[Mesh] OR "Geriatric Assessment"[Mesh] OR "Elder Nutritional Physiological Phenomena"[Mesh] OR "Elder Abuse"[Mesh] OR "Nurses Improving Care for Health System Elders"[Mesh] OR "elderly" [tiab] OR "eldest" [tiab] OR "late-life*" [tiab] OR "frail*" [tiab] OR "geriatri*" [tiab] OR "psychogeriatr*"[tiab] OR "gerontolog*"[tiab] OR "old age*"[tiab] OR "senior*"[tiab] OR "senium"[tiab] OR "very old*"[tiab] OR "septuagenarian*"[tiab] OR "octagenarian*"[tiab] OR "octogenarian*"[tiab] OR "nonagenarian*"[tiab] OR "centarian*"[tiab] OR "centenarian*"[tiab] OR "supercentenarian*"[tiab] OR "older people"[tiab] OR "older subject*"[tiab] OR "older patient*"[tiab] OR "older age*"[tiab] OR "older adult*"[tiab] OR "older man"[tiab] OR "older men"[tiab] OR "older male*"[tiab] OR "older woman"[tiab] OR "older women"[tiab] OR "older female*"[tiab] OR "older population*"[tiab] OR "older person*"[tiab] OR "older individual*"[tiab] OR "aged 55"[tiab] OR "aged 60"[tiab] OR "aged 70"[tiab] OR "aged 80"[tiab] OR "aged 90"[tiab] OR "aged 100"[tiab] OR "over 55"[tiab] OR "over 60"[tiab] OR "over 70"[tiab] OR "over 80"[tiab] OR "over 90"[tiab] OR "over 100"[tiab] OR "55 years and older"[tiab] OR "60 years and older"[tiab] OR "70 years and older"[tiab] OR "80 years and older"[tiab] OR "90 years and older"[tiab] OR "100 years and older"[tiab] OR "55 years and over"[tiab] OR "60 years and over"[tiab] OR "70 years and over"[tiab] OR "80 years and over"[tiab] OR "90 years and over"[tiab] OR "100 years and over"[tiab] OR "55 year and older"[tiab] OR "60 year and older"[tiab] OR "70 year and older"[tiab] OR "80 year and older"[tiab] OR "90 year and older"[tiab] OR "100 year and older"[tiab] OR "55 years or older"[tiab] OR "60 years or older"[tiab] OR "70 years or older"[tiab] OR "80 years or older"[tiab] OR "90 years or older"[tiab] OR "100 years or older"[tiab] OR "55 years or over"[tiab] OR "60 years or over"[tiab] OR "70 years or over"[tiab] OR "80 years or over"[tiab] OR "90 years or over"[tiab] OR "100 years or over"[tiab] OR "55 year or older"[tiab] OR "60 year or older"[tiab] OR "70 year or older"[tiab] OR "80 year or older"[tiab] OR "90 year or older"[tiab] OR "100 year or older"[tiab] OR "older than 55"[tiab] OR "older than 60"[tiab] OR "older than 70"[tiab] OR "older than 80"[tiab] OR "older than 90"[tiab] OR "older than 100"[tiab]** | **3,553,509** |
| **#2** | **"Rehabilitation Centers"[Mesh] OR "Nursing Homes"[Mesh] OR "Housing for the Elderly"[Mesh] OR "Homes for the Aged" [Mesh] OR "Senior Centers"[Mesh] OR "Rehabilitation Center*"[tiab] OR "Rehabilitation Centre*"[tiab] OR "rehab center*"[tiab] OR "rehab centre*"[tiab] OR "Rehabilitation facilit*"[tiab] OR "rehab facilit*"[tiab] OR "nursing home*"[tiab] OR "housing for the elderly"[tiab] OR "homes for the aged"[tiab] OR "senior center*"[tiab] OR "senior centre*"[tiab] OR "skilled nursing facility*"[tiab] OR ("rehabilitat*" AND "hospital*")** | **226,479** |
| **#1** | **"Rehabilitation"[Mesh] OR "rehabilitation"[Subheading] OR "Rehabilitation Nursing"[Mesh] OR "Physical Therapy Modalities"[Mesh] OR "Exercise"[Mesh] OR "Exercise Movement Techniques"[Mesh] OR "Exercise Therapy"[Mesh] OR "Physical Therapy (Specialty)"[MeSH] OR "Psychotherapy"[Mesh] OR "Complementary Therapies"[Mesh] OR "Nutrition Therapy"[Mesh] OR "Health Education"[Mesh] OR "Self-Help Devices"[Mesh] OR "Orthopedic Equipment"[Mesh] OR "rehabilitat*"[tiab] OR "physiotherap*"[tiab] OR ("physical"[tiab] AND ("therapy"[tiab] OR "therapies"[tiab] OR "activity"[tiab] OR "activities"[tiab])) OR "exercis*"[tiab] OR "training*"[tiab] OR ("occupational"[tiab] AND ("therapy"[tiab] OR "therapies"[tiab])) OR "ms-nursing"[tiab] OR "speech therap*"[tiab] OR "language therap*"[tiab] OR "language training*"[tiab] OR "psychotherap*"[tiab] OR "logotherap*"[tiab] OR "art therap*"[tiab] OR "behavior therap*"[tiab] OR "behaviour therap*"[tiab] OR "behavioral therap*"[tiab] OR "behavioural therap*"[tiab] OR "biofeedback*"[tiab] OR "feedback*"[tiab] OR "myofeedback*"[tiab] OR "yoga*"[tiab] OR "complementar*"[tiab] OR "acupunctur*"[tiab] OR "electroacupunctur*"[tiab] OR "kinesio*"[tiab] OR "chiropra*"[tiab] OR "osteopath*"’[tiab] OR "dance therap*"[tiab] OR "music therap*"[tiab] OR "cognitive therap*"[tiab] OR "cbt"[tiab] OR "cognition therap*"[tiab] OR "relaxation*"[tiab] OR "meditati*"[tiab] OR "chronotherap*"[tiab] OR "health educati*"[tiab] OR "patient educati*"[tiab] OR "patients educati*"[tiab] OR "health litera*"[tiab] OR "health informati*"[tiab] OR "energy management*"[tiab] OR "fatigue management*"[tiab] OR "wheel chair*"[tiab] OR "wheelchair*"[tiab] OR "orthos*"[tiab] OR "orthot*"[tiab] OR "brace"[tiab] OR "braces"[tiab] OR "cane"[tiab] OR "canes"[tiab] OR "crutch*"[tiab] OR "walker"[tiab] OR "walkers"[tiab] OR "mindfulness*"[tiab] OR "mirror therap*"[tiab]** | **2,612,316** |

## Table 2: Search strategy in Embase.com

| **Search** | **Query** | **Results** |
| --- | --- | --- |
| **#7** | **#1 AND #2 AND #3 AND #4 AND #5 AND #6** | **1,071** |
| **#6** | 'rehabilitation'/exp OR 'exercise'/exp OR 'physiotherapy'/exp OR 'psychotherapy'/exp OR 'diet therapy'/exp OR 'patient education'/exp OR 'rehabilitation equipment'/exp OR 'orthopedic equipment'/exp OR (‘rehabilitat*’ OR ‘physiotherap*’ OR (‘physical’ AND (‘therapy’ OR ‘therapies’ OR ‘activity’ OR ‘activities’)) OR ‘exercis*’ OR ‘training*’ OR (‘occupational’ AND (‘therapy’ OR ‘therapies’)) OR ‘ms-nursing’ OR ‘speech therap*’ OR ‘language therap*’ OR ‘language training*’ OR ‘psychotherap*’ OR ‘logotherap*’ OR ‘art therap*’ OR ‘behavior therap*’ OR ‘behaviour therap*’ OR ‘behavioral therap*’ OR ‘behavioural therap*’ OR ‘biofeedback*’ OR ‘feedback*’ OR ‘myofeedback*’ OR ‘yoga*’ OR ‘complementar*’ OR ‘acupunctur*’ OR ‘electroacupunctur*’ OR ‘kinesio*’ OR ‘chiropra*’ OR ‘osteopath*’ OR ‘dance therap*’ OR ‘music therap*’ OR ‘cognitive therap*’ OR ‘cbt’ OR ‘cognition therap*’ OR ‘relaxation*’ OR ‘meditati*’ OR ‘chronotherap*’ OR ‘health educati*’ OR ‘patient educati*’ OR ‘patients educati*’ OR ‘health litera*’ OR ‘health informati*’ OR ‘energy management*’ OR ‘fatigue management*’ OR ‘wheel chair*’ OR ‘wheelchair*’ OR ‘orthos*’ OR ‘orthot*’ OR ‘brace’ OR ‘braces’ OR ‘cane’ OR ‘canes’ OR ‘crutch*’ OR ‘walker’ OR ‘walkers’ OR ‘mindfulness*’ OR ‘mirror therap*’):ti,ab,kw | **3,381,599** |
| **#5** | 'rehabilitation center'/exp OR 'nursing home'/exp OR 'elderly care'/exp OR 'senior center'/exp OR (‘Rehabilitation Center*’ OR ‘Rehabilitation Centre*’ OR ‘rehab center*’ OR ‘rehab centre*’ OR ‘Rehabilitation facilit*’ OR ‘rehab facilit*’ OR ‘nursing home*’ OR ‘housing for the elderly’ OR ‘homes for the aged’ OR ‘senior center*’ OR ‘senior centre*’ OR ‘skilled nursing facility*’ OR (‘rehabilitat*’ AND ‘hospital*’)):ti,ab,kw | **202,638** |
| **#4** | 'aged'/exp OR 'geriatrics'/exp OR 'geriatrician'/exp OR 'elderly care'/exp OR 'retirement'/exp OR 'geriatric assessment'/exp OR 'geriatric nutrition'/exp OR (‘elderly’ OR ‘eldest’ OR ‘late-life*’ OR ‘frail*’ OR ‘geriatri*’ OR ‘psychogeriatr*’ OR ‘gerontolog*’ OR ‘old age*’ OR ‘senior*’ OR ‘senium’ OR ‘very old*’ OR ‘septuagenarian*’ OR ‘octagenarian*’ OR ‘octogenarian*’ OR ‘nonagenarian*’ OR ‘centarian*’ OR ‘centenarian*’ OR ‘supercentenarian*’ OR ‘older people’ OR ‘older subject*’ OR ‘older patient*’ OR ‘older age*’ OR ‘older adult*’ OR ‘older man’ OR ‘older men’ OR ‘older male*’ OR ‘older woman’ OR ‘older women’ OR ‘older female*’ OR ‘older population*’ OR ‘older person*’ OR ‘older individual*’ OR ‘aged 55’ OR ‘aged 60’ OR ‘aged 70’ OR ‘aged 80’ OR ‘aged 90’ OR ‘aged 100’ OR ‘over 55’ OR ‘over 60’ OR ‘over 70’ OR ‘over 80’ OR ‘over 90’ OR ‘over 100’ OR ‘55 years and older’ OR ‘60 years and older’ OR ‘70 years and older’ OR ‘80 years and older’ OR ‘90 years and older’ OR ‘100 years and older’ OR ‘55 years and over’ OR ‘60 years and over’ OR ‘70 years and over’ OR ‘80 years and over’ OR ‘90 years and over’ OR ‘100 years and over’ OR ‘55 year and older’ OR ‘60 year and older’ OR ‘70 year and older’ OR ‘80 year and older’ OR ‘90 year and older’ OR ‘100 year and older’ OR ‘55 years or older’ OR ‘60 years or older’ OR ‘70 years or older’ OR ‘80 years or older’ OR ‘90 years or older’ OR ‘100 years or older’ OR ‘55 years or over’ OR ‘60 years or over’ OR ‘70 years or over’ OR ‘80 years or over’ OR ‘90 years or over’ OR ‘100 years or over’ OR ‘55 year or older’ OR ‘60 year or older’ OR ‘70 year or older’ OR ‘80 year or older’ OR ‘90 year or older’ OR ‘100 year or older’ OR ‘older than 55’ OR ‘older than 60’ OR ‘older than 70’ OR ‘older than 80’ OR ‘older than 90’ OR ‘older than 100’):ti,ab,kw | **3,723,791** |
| **#3** | (‘quality indicator*’ OR ‘Health Metric*’ OR ‘quality of rehabilitation*’ OR ‘function activity and participation’ OR ‘international classification of functioning’ OR ‘ICF model’):ti,ab,kw OR 'quality of life'/exp OR (life NEXT/1 qualit*):ti,ab,kw OR 'quality of life':ti,ab,kw OR 'daily life activity'/exp OR 'activities of daily living':ti,ab,kw OR ('daily living' NEXT/1 activit*):ti,ab,kw OR ('daily live' NEXT/1 activit*):ti,ab,kw OR 'adl':ti,ab,kw OR 'chronic limitation of activity':ti,ab,kw OR (self NEXT/1 care*):ti,ab,kw OR 'health status'/exp OR 'health status':ti,ab,kw OR 'level of health':ti,ab,kw OR (health NEXT/1 level*):ti,ab,kw OR 'qol':ti,ab,kw OR 'hrql':ti,ab,kw OR 'hrqol':ti,ab,kw OR ‘time of discharge’:ti,ab,kw OR ‘discharge time’:ti,ab,kw | **1,014,780** |
| **#2** | (‘patient perspective*’ OR ‘patient s perspective*’ OR ‘patient centred’ OR ‘patient centered’ OR ‘experience*’ OR ‘belief*’ OR ‘satisf*’ OR ‘attitude*’ OR ‘expectation*’ OR ‘reaction*’ OR ‘opinion*’ OR ‘acceptance’ OR ‘view*’ OR ‘perception*’ OR ‘concern*’ OR ‘self report*’):ti,ab,kw | **5,472,640** |
| **#1** | 'qualitative research'/exp OR 'interview'/exp OR 'observational study'/exp OR 'tape recorder'/exp OR 'grounded theory'/exp OR (‘thematic analys*’ OR ‘content analys*’ OR ‘focus group*’ OR ‘ethnograph*’ OR ‘ethnograf*’ OR ‘etnograf*’ OR ‘field stud*’ OR ‘phenomenolog*’ OR ‘narration*’ OR ‘narrative’ ‘case stud*’ OR ‘qualitative stud*’ OR ‘qualitative analys*’ OR ‘qualitative research*’ OR ‘qualitative method*’ OR ‘multimethodolog*’ OR ‘mixed method*’ OR ‘observation*’ OR ‘grounded theor*’ OR ‘audio recording*’ OR ‘tape recording*’ OR ‘audiotape*’ OR ((‘semi-structured’ OR ‘semistructured’ OR ‘unstructured’ OR ‘informal’ OR ‘in-depth’ OR ‘indepth’ OR ‘face-to-face’ OR ‘structured’ OR ‘guide*’) AND (‘interview*’ OR ‘discussion*’ OR ‘questionnaire*’))):ti,ab,kw | **1,811,396** |

## Table 3: Search strategy in Clarivate Analytics/Web of Science Core Collection

| **Search** | **Query** | **Results** |
| --- | --- | --- |
| **#7** | **#1 AND #2 AND #3 AND #4 AND #5 AND #6** | **267** |
| **#6** | TS=(“rehabilitat*” OR “physiotherap*” OR (“physical” AND (“therapy” OR “therapies” OR “activity” OR “activities”)) OR “exercis*” OR “training*” OR (“occupational” AND (“therapy” OR “therapies”)) OR "ms-nursing" OR “speech therap*” OR “language therap*” OR “language training*” OR “psychotherap*” OR “logotherap*” OR “art therap*” OR “behavior therap*” OR “behaviour therap*” OR “behavioral therap*” OR “behavioural therap*” OR “biofeedback*” OR “feedback*” OR “myofeedback*” OR “yoga*” OR “complementar*” OR “acupunctur*” OR “electroacupunctur*” OR “kinesio*” OR “chiropra*” OR “osteopath*”’ OR “dance therap*” OR “music therap*” OR “cognitive therap*” OR “cbt” OR “cognition therap*” OR “relaxation*” OR “meditati*” OR “chronotherap*” OR “health educati*” OR “patient educati*” OR “patients educati*” OR “health litera*” OR “health informati*” OR “energy management*” OR “fatigue management*” OR “wheel chair*” OR “wheelchair*” OR “orthos*” OR “orthot*” OR “brace” OR “braces” OR “cane” OR “canes” OR “crutch*” OR “walker” OR “walkers” OR “mindfulness*” OR “mirror therap*”) | **2,849,108** |
| **#5** | TS=("Rehabilitation Center*" OR "Rehabilitation Centre*" OR “rehab center*” OR “rehab centre*” OR "Rehabilitation facilit*" OR “rehab facilit*” OR “nursing home*” OR “housing for the elderly” OR “homes for the aged” OR “senior center*” OR “senior centre*” OR “skilled nursing facility*” OR (“rehabilitat*” AND “hospital*”)) | **75,223** |
| **#4** | TS=(“elderly” OR “eldest” OR “late-life*” OR “frail*” OR “geriatri*” OR “psychogeriatr*” OR “gerontolog*” OR “old age*” OR “senior*” OR “senium” OR “very old*” OR “septuagenarian*” OR “octagenarian*” OR “octogenarian*” OR “nonagenarian*” OR “centarian*” OR “centenarian*” OR “supercentenarian*” OR “older people” OR “older subject*” OR “older patient*” OR “older age*” OR “older adult*” OR “older man” OR “older men” OR “older male*” OR “older woman” OR “older women” OR “older female*” OR “older population*” OR “older person*” OR “older individual*” OR "aged 55" OR "aged 60" OR "aged 70" OR "aged 80" OR "aged 90" OR "aged 100" OR "over 55" OR "over 60" OR "over 70" OR "over 80" OR "over 90" OR "over 100" OR "55 years and older" OR "60 years and older" OR "70 years and older" OR "80 years and older" OR "90 years and older" OR "100 years and older" OR "55 years and over" OR "60 years and over" OR "70 years and over" OR "80 years and over" OR "90 years and over" OR "100 years and over" OR "55 year and older" OR "60 year and older" OR "70 year and older" OR "80 year and older" OR "90 year and older" OR "100 year and older" OR "55 years or older" OR "60 years or older" OR "70 years or older" OR "80 years or older" OR "90 years or older" OR "100 years or older" OR "55 years or over" OR "60 years or over" OR "70 years or over" OR "80 years or over" OR "90 years or over" OR "100 years or over" OR "55 year or older" OR "60 year or older" OR "70 year or older" OR "80 year or older" OR "90 year or older" OR "100 year or older" OR "older than 55" OR "older than 60" OR "older than 70" OR "older than 80" OR "older than 90" OR "older than 100") | **787,873** |
| **#3** | TS=(“quality indicator*” OR “Health Metric*” OR “quality of rehabilitation*” OR “function activity and participation” OR “international classification of functioning” OR “ICF model” OR "quality of life" OR “life qualit*” OR “living qualit*” OR "quality of living" OR "activities of daily living" OR "activity of daily living" OR "activities of daily life" OR "activity of daily life" OR “daily living activit*” OR “daily life activit*” OR "adl" OR "chronic limitation of activity" OR “self care*” OR "health status" OR "level of health" OR “health level*” OR "qol" OR "hrql" OR "hrqol" OR “time of discharge” OR “discharge time”) | **546,682** |
| **#2** | TS=(“patient perspective*” OR “patient s perspective*” OR “patient centred” OR “patient centered” OR “experience*” OR “belief*” OR “satisf*” OR “attitude*” OR “expectation*” OR “reaction*” OR “opinion*” OR “acceptance” OR “view*” OR “perception*” OR “concern*” OR “self report*”) | **7,918,583** |
| **#1** | TS=(“thematic analys*” OR “content analys*” OR “focus group*” OR “ethnograph*” OR “ethnograf*” OR “etnograf*” OR “field stud*” OR “phenomenolog*” OR “narration*” OR “narrative” “case stud*” OR “qualitative stud*” OR “qualitative analys*” OR “qualitative research*” OR “qualitative method*” OR “multimethodolog*” OR “mixed method*” OR “observation*” OR “grounded theor*” OR “audio recording*” OR “tape recording*” OR “audiotape*” OR ((“semi-structured” OR “semistructured” OR “unstructured” OR “informal” OR “in-depth” OR “indepth” OR “face-to-face” OR “structured” OR “guide*”) AND (“interview*” OR “discussion*” OR “questionnaire*”))) | **2,319,526** |

## Table 4: Search strategy in Cumulative Index to Nursing and Allied Health Literature (CINAHL)

| **Search** | **Query** | **Results** |
| --- | --- | --- |
| **#7** | **#1 AND #2 AND #3 AND #4 AND #5 AND #6** | **716** |
| **#6** | MH ("Qualitative Studies+" OR "Thematic Analysis" OR "Content Analysis" OR "Focus Groups" OR "Ethnographic Research" OR "Field Studies" OR "Narratives+" OR "Multimethod Studies" OR "Observational Methods+" OR "Audiorecording" OR "Semi-Structured Interview" OR "Unstructured Interview") OR TI(“thematic analys*” OR “content analys*” OR “focus group*” OR ethnograph* OR ethnograf* OR etnograf* OR “field stud*” OR phenomenolog* OR narration* OR narrative OR “qualitative stud*” OR “qualitative analys*” OR “qualitative research*” OR “qualitative method*” OR multimethodolog* OR “mixed method*” OR observation* OR “grounded theory” OR “audio recording*” OR “tape recording*” OR audiotape* OR ((“semi-structured” OR semistructured OR unstructured OR informal OR “in-depth” OR indepth OR “face-to-face” OR structured OR guide*) AND (interview* OR discussion* OR questionnaire*))) OR AB(“thematic analys*” OR “content analys*” OR “focus group*” OR ethnograph* OR ethnograf* OR etnograf* OR “field stud*” OR phenomenolog* OR narration* OR narrative OR “qualitative stud*” OR “qualitative analys*” OR “qualitative research*” OR “qualitative method*” OR multimethodolog* OR “mixed method*” OR observation* OR “grounded theory” OR “audio recording*” OR “tape recording*” OR audiotape* OR ((“semi-structured” OR semistructured OR unstructured OR informal OR “in-depth” OR indepth OR “face-to-face” OR structured OR guide*) AND (interview* OR discussion* OR questionnaire*))) OR KW(“thematic analys*” OR “content analys*” OR “focus group*” OR ethnograph* OR ethnograf* OR etnograf* OR “field stud*” OR phenomenolog* OR narration* OR narrative OR “qualitative stud*” OR “qualitative analys*” OR “qualitative research*” OR “qualitative method*” OR multimethodolog* OR “mixed method*” OR observation* OR “grounded theory” OR “audio recording*” OR “tape recording*” OR audiotape* OR ((“semi-structured” OR semistructured OR unstructured OR informal OR “in-depth” OR indepth OR “face-to-face” OR structured OR guide*) AND (interview* OR discussion* OR questionnaire*))) | **511,844** |
| **#5** | TI(“patient perspective*” OR “patient s perspective*” OR “patient centred” OR “patient centered” OR “experience*” OR “belief*” OR “satisf*” OR “attitude*” OR “expectation*” OR “reaction*” OR “opinion*” OR “acceptance” OR “view*” OR “perception*” OR “concern*” OR “self report*”) OR AB(“patient perspective*” OR “patient s perspective*” OR “patient centred” OR “patient centered” OR “experience*” OR “belief*” OR “satisf*” OR “attitude*” OR “expectation*” OR “reaction*” OR “opinion*” OR “acceptance” OR “view*” OR “perception*” OR “concern*” OR “self report*”) OR KW(“patient perspective*” OR “patient s perspective*” OR “patient centred” OR “patient centered” OR “experience*” OR “belief*” OR “satisf*” OR “attitude*” OR “expectation*” OR “reaction*” OR “opinion*” OR “acceptance” OR “view*” OR “perception*” OR “concern*” OR “self report*”) | **1,112,755** |
| **#4** | (MH "Quality of Health Care+") OR (MH "Quality of Care Research") OR (MH "Quality of Life+") OR (MH "Activities of Daily Living+") OR (MH "Health Status+") OR TI(“quality indicator*” OR “Health Metric*” OR “quality of rehabilitation*” OR “function activity and participation” OR “international classification of functioning” OR “ICF model” OR OR "quality of life" OR “life qualit*” OR “living qualit*” OR "quality of living" OR "activities of daily living" OR "activity of daily living" OR "activities of daily life" OR "activity of daily life" OR “daily living activit*” OR “daily life activit*” OR "adl" OR "chronic limitation of activity" OR “self care*” OR "health status" OR "level of health" OR “health level*” OR "qol" OR "hrql" OR "hrqol" OR “time of discharge” OR “discharge time”) OR AB(“quality indicator*” OR “Health Metric*” OR “quality of rehabilitation*” OR “function activity and participation” OR “international classification of functioning” OR “ICF model” OR OR "quality of life" OR “life qualit*” OR “living qualit*” OR "quality of living" OR "activities of daily living" OR "activity of daily living" OR "activities of daily life" OR "activity of daily life" OR “daily living activit*” OR “daily life activit*” OR "adl" OR "chronic limitation of activity" OR “self care*” OR "health status" OR "level of health" OR “health level*” OR "qol" OR "hrql" OR "hrqol" OR “time of discharge” OR “discharge time”) OR KW(“quality indicator*” OR “Health Metric*” OR “quality of rehabilitation*” OR “function activity and participation” OR “international classification of functioning” OR “ICF model” OR OR "quality of life" OR “life qualit*” OR “living qualit*” OR "quality of living" OR "activities of daily living" OR "activity of daily living" OR "activities of daily life" OR "activity of daily life" OR “daily living activit*” OR “daily life activit*” OR "adl" OR "chronic limitation of activity" OR “self care*” OR "health status" OR "level of health" OR “health level*” OR "qol" OR "hrql" OR "hrqol" OR “time of discharge” OR “discharge time”) | **1,088,998** |
| **#3** | (MH "Aged") OR (MH "Geriatrics") OR (MH "Geriatricians") OR (MH "Geriatric Psychiatry") OR (MH "Gerontologic Nursing+") OR (MH "Health Services for the Aged") OR (MH "Retirement") OR (MH "Geriatric Nutritional Physiology") OR  TI(“elderly” [tiab] OR “eldest” [tiab] OR “late-life*” [tiab] OR “frail*” [tiab] OR “geriatri*” [tiab] OR “psychogeriatr*”[tiab] OR “gerontolog*”[tiab] OR “old age*”[tiab] OR “senior*”[tiab] OR “senium”[tiab] OR “very old*”[tiab] OR “septuagenarian*”[tiab] OR “octagenarian*”[tiab] OR “octogenarian*”[tiab] OR “nonagenarian*”[tiab] OR “centarian*”[tiab] OR “centenarian*”[tiab] OR “supercentenarian*”[tiab] OR “older people”[tiab] OR “older subject*”[tiab] OR “older patient*”[tiab] OR “older age*”[tiab] OR “older adult*”[tiab] OR “older man”[tiab] OR “older men”[tiab] OR “older male*”[tiab] OR “older woman”[tiab] OR “older women”[tiab] OR “older female*”[tiab] OR “older population*”[tiab] OR “older person*”[tiab] OR “older individual*”[tiab] OR "aged 55"[tiab] OR "aged 60"[tiab] OR "aged 70"[tiab] OR "aged 80"[tiab] OR "aged 90"[tiab] OR "aged 100"[tiab] OR "over 55"[tiab] OR "over 60"[tiab] OR "over 70"[tiab] OR "over 80"[tiab] OR "over 90"[tiab] OR "over 100"[tiab] OR "55 years and older"[tiab] OR "60 years and older"[tiab] OR "70 years and older"[tiab] OR "80 years and older"[tiab] OR "90 years and older"[tiab] OR "100 years and older"[tiab] OR "55 years and over"[tiab] OR "60 years and over"[tiab] OR "70 years and over"[tiab] OR "80 years and over"[tiab] OR "90 years and over"[tiab] OR "100 years and over"[tiab] OR "55 year and older"[tiab] OR "60 year and older"[tiab] OR "70 year and older"[tiab] OR "80 year and older"[tiab] OR "90 year and older"[tiab] OR "100 year and older"[tiab] OR "55 years or older"[tiab] OR "60 years or older"[tiab] OR "70 years or older"[tiab] OR "80 years or older"[tiab] OR "90 years or older"[tiab] OR "100 years or older"[tiab] OR "55 years or over"[tiab] OR "60 years or over"[tiab] OR "70 years or over"[tiab] OR "80 years or over"[tiab] OR "90 years or over"[tiab] OR "100 years or over"[tiab] OR "55 year or older"[tiab] OR "60 year or older"[tiab] OR "70 year or older"[tiab] OR "80 year or older"[tiab] OR "90 year or older"[tiab] OR "100 year or older"[tiab] OR "older than 55"[tiab] OR "older than 60"[tiab] OR "older than 70"[tiab] OR "older than 80"[tiab] OR "older than 90"[tiab] OR "older than 100"[tiab]) OR AB(“elderly” [tiab] OR “eldest” [tiab] OR “late-life*” [tiab] OR “frail*” [tiab] OR “geriatri*” [tiab] OR “psychogeriatr*”[tiab] OR “gerontolog*”[tiab] OR “old age*”[tiab] OR “senior*”[tiab] OR “senium”[tiab] OR “very old*”[tiab] OR “septuagenarian*”[tiab] OR “octagenarian*”[tiab] OR “octogenarian*”[tiab] OR “nonagenarian*”[tiab] OR “centarian*”[tiab] OR “centenarian*”[tiab] OR “supercentenarian*”[tiab] OR “older people”[tiab] OR “older subject*”[tiab] OR “older patient*”[tiab] OR “older age*”[tiab] OR “older adult*”[tiab] OR “older man”[tiab] OR “older men”[tiab] OR “older male*”[tiab] OR “older woman”[tiab] OR “older women”[tiab] OR “older female*”[tiab] OR “older population*”[tiab] OR “older person*”[tiab] OR “older individual*”[tiab] OR "aged 55"[tiab] OR "aged 60"[tiab] OR "aged 70"[tiab] OR "aged 80"[tiab] OR "aged 90"[tiab] OR "aged 100"[tiab] OR "over 55"[tiab] OR "over 60"[tiab] OR "over 70"[tiab] OR "over 80"[tiab] OR "over 90"[tiab] OR "over 100"[tiab] OR "55 years and older"[tiab] OR "60 years and older"[tiab] OR "70 years and older"[tiab] OR "80 years and older"[tiab] OR "90 years and older"[tiab] OR "100 years and older"[tiab] OR "55 years and over"[tiab] OR "60 years and over"[tiab] OR "70 years and over"[tiab] OR "80 years and over"[tiab] OR "90 years and over"[tiab] OR "100 years and over"[tiab] OR "55 year and older"[tiab] OR "60 year and older"[tiab] OR "70 year and older"[tiab] OR "80 year and older"[tiab] OR "90 year and older"[tiab] OR "100 year and older"[tiab] OR "55 years or older"[tiab] OR "60 years or older"[tiab] OR "70 years or older"[tiab] OR "80 years or older"[tiab] OR "90 years or older"[tiab] OR "100 years or older"[tiab] OR "55 years or over"[tiab] OR "60 years or over"[tiab] OR "70 years or over"[tiab] OR "80 years or over"[tiab] OR "90 years or over"[tiab] OR "100 years or over"[tiab] OR "55 year or older"[tiab] OR "60 year or older"[tiab] OR "70 year or older"[tiab] OR "80 year or older"[tiab] OR "90 year or older"[tiab] OR "100 year or older"[tiab] OR "older than 55"[tiab] OR "older than 60"[tiab] OR "older than 70"[tiab] OR "older than 80"[tiab] OR "older than 90"[tiab] OR "older than 100"[tiab]) OR KW(“elderly” [tiab] OR “eldest” [tiab] OR “late-life*” [tiab] OR “frail*” [tiab] OR “geriatri*” [tiab] OR “psychogeriatr*”[tiab] OR “gerontolog*”[tiab] OR “old age*”[tiab] OR “senior*”[tiab] OR “senium”[tiab] OR “very old*”[tiab] OR “septuagenarian*”[tiab] OR “octagenarian*”[tiab] OR “octogenarian*”[tiab] OR “nonagenarian*”[tiab] OR “centarian*”[tiab] OR “centenarian*”[tiab] OR “supercentenarian*”[tiab] OR “older people”[tiab] OR “older subject*”[tiab] OR “older patient*”[tiab] OR “older age*”[tiab] OR “older adult*”[tiab] OR “older man”[tiab] OR “older men”[tiab] OR “older male*”[tiab] OR “older woman”[tiab] OR “older women”[tiab] OR “older female*”[tiab] OR “older population*”[tiab] OR “older person*”[tiab] OR “older individual*”[tiab] OR "aged 55"[tiab] OR "aged 60"[tiab] OR "aged 70"[tiab] OR "aged 80"[tiab] OR "aged 90"[tiab] OR "aged 100"[tiab] OR "over 55"[tiab] OR "over 60"[tiab] OR "over 70"[tiab] OR "over 80"[tiab] OR "over 90"[tiab] OR "over 100"[tiab] OR "55 years and older"[tiab] OR "60 years and older"[tiab] OR "70 years and older"[tiab] OR "80 years and older"[tiab] OR "90 years and older"[tiab] OR "100 years and older"[tiab] OR "55 years and over"[tiab] OR "60 years and over"[tiab] OR "70 years and over"[tiab] OR "80 years and over"[tiab] OR "90 years and over"[tiab] OR "100 years and over"[tiab] OR "55 year and older"[tiab] OR "60 year and older"[tiab] OR "70 year and older"[tiab] OR "80 year and older"[tiab] OR "90 year and older"[tiab] OR "100 year and older"[tiab] OR "55 years or older"[tiab] OR "60 years or older"[tiab] OR "70 years or older"[tiab] OR "80 years or older"[tiab] OR "90 years or older"[tiab] OR "100 years or older"[tiab] OR "55 years or over"[tiab] OR "60 years or over"[tiab] OR "70 years or over"[tiab] OR "80 years or over"[tiab] OR "90 years or over"[tiab] OR "100 years or over"[tiab] OR "55 year or older"[tiab] OR "60 year or older"[tiab] OR "70 year or older"[tiab] OR "80 year or older"[tiab] OR "90 year or older"[tiab] OR "100 year or older"[tiab] OR "older than 55"[tiab] OR "older than 60"[tiab] OR "older than 70"[tiab] OR "older than 80"[tiab] OR "older than 90"[tiab] OR "older than 100"[tiab]) | **857,303** |
| **#2** | (MH "Rehabilitation Centers+") OR (MH "Nursing Homes+") OR (MH "Nursing Home Patients") OR (MH "Housing for the Elderly") OR (MH "Senior Centers") OR TI("Rehabilitation Center*" OR "Rehabilitation Centre*" OR “rehab center*” OR “rehab centre*” OR "Rehabilitation facilit*" OR “rehab facilit*” OR “nursing home*” OR “housing for the elderly” OR “homes for the aged” OR “senior center*” OR “senior centre*” OR “skilled nursing facility*” OR (“rehabilitat*” AND “hospital*”)) OR AB("Rehabilitation Center*" OR "Rehabilitation Centre*" OR “rehab center*” OR “rehab centre*” OR "Rehabilitation facilit*" OR “rehab facilit*” OR “nursing home*” OR “housing for the elderly” OR “homes for the aged” OR “senior center*” OR “senior centre*” OR “skilled nursing facility*” OR (“rehabilitat*” AND “hospital*”)) OR KW("Rehabilitation Center*" OR "Rehabilitation Centre*" OR “rehab center*” OR “rehab centre*” OR "Rehabilitation facilit*" OR “rehab facilit*” OR “nursing home*” OR “housing for the elderly” OR “homes for the aged” OR “senior center*” OR “senior centre*” OR “skilled nursing facility*” OR (“rehabilitat*” AND “hospital*”)) | **72,307** |
| **#1** | (MH "Rehabilitation+") OR (MH "Rehabilitation Nursing") OR (MH "Physical Therapy+") OR (MH "Exercise+") OR (MH "Physical Therapy+") OR (MH "Psychotherapy+") OR (MH "Health Education+") OR (MH "Orthopedic Equipment and Supplies") OR TI(“rehabilitat*” OR “physiotherap*” OR (“physical” AND (“therapy” OR “therapies” OR “activity” OR “activities”)) OR “exercis*” OR “training*” OR (“occupational” AND (“therapy” OR “therapies”)) OR "ms-nursing" OR “speech therap*” OR “language therap*” OR “language training*” OR “psychotherap*” OR “logotherap*” OR “art therap*” OR “behavior therap*” OR “behaviour therap*” OR “behavioral therap*” OR “behavioural therap*” OR “biofeedback*” OR “feedback*” OR “myofeedback*” OR “yoga*” OR “complementar*” OR “acupunctur*” OR “electroacupunctur*” OR “kinesio*” OR “chiropra*” OR “osteopath*”’ OR “dance therap*” OR “music therap*” OR “cognitive therap*” OR “cbt” OR “cognition therap*” OR “relaxation*” OR “meditati*” OR “chronotherap*” OR “health educati*” OR “patient educati*” OR “patients educati*” OR “health litera*” OR “health informati*” OR “energy management*” OR “fatigue management*” OR “wheel chair*” OR “wheelchair*” OR “orthos*” OR “orthot*” OR “brace” OR “braces” OR “cane” OR “canes” OR “crutch*” OR “walker” OR “walkers” OR “mindfulness*” OR “mirror therap*”) OR AB(“rehabilitat*” OR “physiotherap*” OR (“physical” AND (“therapy” OR “therapies” OR “activity” OR “activities”)) OR “exercis*” OR “training*” OR (“occupational” AND (“therapy” OR “therapies”)) OR "ms-nursing" OR “speech therap*” OR “language therap*” OR “language training*” OR “psychotherap*” OR “logotherap*” OR “art therap*” OR “behavior therap*” OR “behaviour therap*” OR “behavioral therap*” OR “behavioural therap*” OR “biofeedback*” OR “feedback*” OR “myofeedback*” OR “yoga*” OR “complementar*” OR “acupunctur*” OR “electroacupunctur*” OR “kinesio*” OR “chiropra*” OR “osteopath*”’ OR “dance therap*” OR “music therap*” OR “cognitive therap*” OR “cbt” OR “cognition therap*” OR “relaxation*” OR “meditati*” OR “chronotherap*” OR “health educati*” OR “patient educati*” OR “patients educati*” OR “health litera*” OR “health informati*” OR “energy management*” OR “fatigue management*” OR “wheel chair*” OR “wheelchair*” OR “orthos*” OR “orthot*” OR “brace” OR “braces” OR “cane” OR “canes” OR “crutch*” OR “walker” OR “walkers” OR “mindfulness*” OR “mirror therap*”) OR KW(“rehabilitat*” OR “physiotherap*” OR (“physical” AND (“therapy” OR “therapies” OR “activity” OR “activities”)) OR “exercis*” OR “training*” OR (“occupational” AND (“therapy” OR “therapies”)) OR "ms-nursing" OR “speech therap*” OR “language therap*” OR “language training*” OR “psychotherap*” OR “logotherap*” OR “art therap*” OR “behavior therap*” OR “behaviour therap*” OR “behavioral therap*” OR “behavioural therap*” OR “biofeedback*” OR “feedback*” OR “myofeedback*” OR “yoga*” OR “complementar*” OR “acupunctur*” OR “electroacupunctur*” OR “kinesio*” OR “chiropra*” OR “osteopath*”’ OR “dance therap*” OR “music therap*” OR “cognitive therap*” OR “cbt” OR “cognition therap*” OR “relaxation*” OR “meditati*” OR “chronotherap*” OR “health educati*” OR “patient educati*” OR “patients educati*” OR “health litera*” OR “health informati*” OR “energy management*” OR “fatigue management*” OR “wheel chair*” OR “wheelchair*” OR “orthos*” OR “orthot*” OR “brace” OR “braces” OR “cane” OR “canes” OR “crutch*” OR “walker” OR “walkers” OR “mindfulness*” OR “mirror therap*”) | **1,110,699** |

## Table 3: Search strategy in Wiley/Cochrane Library

| **Search** | **Query** | **Results** |
| --- | --- | --- |
| **#7** | **#1 AND #2 AND #3 AND #4 AND #5 AND #6** | **23** |
| **#6** | (“rehabilitat*” OR “physiotherap*” OR (“physical” AND (“therapy” OR “therapies” OR “activity” OR “activities”)) OR “exercis*” OR “training*” OR (“occupational” AND (“therapy” OR “therapies”)) OR "ms-nursing" OR “speech therap*” OR “language therap*” OR “language training*” OR “psychotherap*” OR “logotherap*” OR “art therap*” OR “behavior therap*” OR “behaviour therap*” OR “behavioral therap*” OR “behavioural therap*” OR “biofeedback*” OR “feedback*” OR “myofeedback*” OR “yoga*” OR “complementar*” OR “acupunctur*” OR “electroacupunctur*” OR “kinesio*” OR “chiropra*” OR “osteopath*”’ OR “dance therap*” OR “music therap*” OR “cognitive therap*” OR “cbt” OR “cognition therap*” OR “relaxation*” OR “meditati*” OR “chronotherap*” OR “health educati*” OR “patient educati*” OR “patients educati*” OR “health litera*” OR “health informati*” OR “energy management*” OR “fatigue management*” OR “wheel chair*” OR “wheelchair*” OR “orthos*” OR “orthot*” OR “brace” OR “braces” OR “cane” OR “canes” OR “crutch*” OR “walker” OR “walkers” OR “mindfulness*” OR “mirror therap*”):ti,ab,kw | **195,290** |
| **#5** | ("Rehabilitation Center*" OR "Rehabilitation Centre*" OR “rehab center*” OR “rehab centre*” OR "Rehabilitation facilit*" OR “rehab facilit*” OR “nursing home*” OR “housing for the elderly” OR “homes for the aged” OR “senior center*” OR “senior centre*” OR “skilled nursing facility*” OR (“rehabilitat*” AND “hospital*”)):ti,ab,kw | **5,447** |
| **#4** | (“elderly” OR “eldest” OR “late-life*” OR “frail*” OR “geriatri*” OR “psychogeriatr*” OR “gerontolog*” OR “old age*” OR “senior*” OR “senium” OR “very old*” OR “septuagenarian*” OR “octagenarian*” OR “octogenarian*” OR “nonagenarian*” OR “centarian*” OR “centenarian*” OR “supercentenarian*” OR “older people” OR “older subject*” OR “older patient*” OR “older age*” OR “older adult*” OR “older man” OR “older men” OR “older male*” OR “older woman” OR “older women” OR “older female*” OR “older population*” OR “older person*” OR “older individual*” OR "aged 55" OR "aged 60" OR "aged 70" OR "aged 80" OR "aged 90" OR "aged 100" OR "over 55" OR "over 60" OR "over 70" OR "over 80" OR "over 90" OR "over 100" OR "55 years and older" OR "60 years and older" OR "70 years and older" OR "80 years and older" OR "90 years and older" OR "100 years and older" OR "55 years and over" OR "60 years and over" OR "70 years and over" OR "80 years and over" OR "90 years and over" OR "100 years and over" OR "55 year and older" OR "60 year and older" OR "70 year and older" OR "80 year and older" OR "90 year and older" OR "100 year and older" OR "55 years or older" OR "60 years or older" OR "70 years or older" OR "80 years or older" OR "90 years or older" OR "100 years or older" OR "55 years or over" OR "60 years or over" OR "70 years or over" OR "80 years or over" OR "90 years or over" OR "100 years or over" OR "55 year or older" OR "60 year or older" OR "70 year or older" OR "80 year or older" OR "90 year or older" OR "100 year or older" OR "older than 55" OR "older than 60" OR "older than 70" OR "older than 80" OR "older than 90" OR "older than 100"):ti,ab,kw | **118,837** |
| **#3** | (“quality indicator*” OR “Health Metric*” OR “quality of rehabilitation*” OR “function activity and participation” OR “international classification of functioning” OR “ICF model” OR "quality of life" OR “life qualit*” OR “living qualit*” OR "quality of living" OR "activities of daily living" OR "activity of daily living" OR "activities of daily life" OR "activity of daily life" OR “daily living activit*” OR “daily life activit*” OR "adl" OR "chronic limitation of activity" OR “self care*” OR "health status" OR "level of health" OR “health level*” OR "qol" OR "hrql" OR "hrqol" OR “time of discharge” OR “discharge time”) :ti,ab,kw | **145,196** |
| **#2** | (“patient perspective*” OR “patient s perspective*” OR “patient centred” OR “patient centered” OR “experience*” OR “belief*” OR “satisf*” OR “attitude*” OR “expectation*” OR “reaction*” OR “opinion*” OR “acceptance” OR “view*” OR “perception*” OR “concern*” OR “self report*”):ti,ab,kw | **227,565** |
| **#1** | (“thematic analys*” OR “content analys*” OR “focus group*” OR “ethnograph*” OR “ethnograf*” OR “etnograf*” OR “field stud*” OR “phenomenolog*” OR “narration*” OR “narrative” “case stud*” OR “qualitative stud*” OR “qualitative analys*” OR “qualitative research*” OR “qualitative method*” OR “multimethodolog*” OR “mixed method*” OR “observation*” OR “grounded theor*” OR “audio recording*” OR “tape recording*” OR “audiotape*” OR ((“semi-structured” OR “semistructured” OR “unstructured” OR “informal” OR “in-depth” OR “indepth” OR “face-to-face” OR “structured” OR “guide*”) AND (“interview*” OR “discussion*” OR “questionnaire*”))):ti,ab,kw | **71,573** |
